# Review on the Application of Biologic Factors in Atopic Dermatitis and Chronic Spontaneous Urticaria

**DOI:** 10.1155/drp/5283760

**Published:** 2026-06-21

**Authors:** Aikaterini Salavoura

**Affiliations:** ^1^ Children’s Hospital ‘Agia Sofia’, First Pediatric Clinic University of Athens, Athens, Greece

**Keywords:** atopic dermatitis, chronic spontaneous urticaria, monoclonal antibodies

## Abstract

**Purpose:**

The use of biologic factors in atopic dermatitis (AD) and chronic spontaneous urticaria (CSU).

**Methods:**

Data from different phase clinical trials, including RCTs and observational and real‐world evidence, which were published in PubMed, were classified according to parameters evaluating AD and CSU. The keywords included the scientific names of different biological factors, AD and CSU.

**Results:**

Recalcitrant cases of AD and chronic CSU are rare, but they impose a burden on the lives of patients and a significant treatment cost. Monoclonal antibodies are currently used to treat a variety of autoimmune skin disorders. However, direct application of previous experience to AD and CSU was not applicable, because AD etiology involves a number of cytokine networks, and CSU seems to be related to a disorganized cytokine background.

**Conclusions:**

Dupilumab is ans FDA‐ and EMA‐approved monoclonal antibody, which is effective in 40%–60% of severe AD cases. FDA/EMA‐approved are also baricitinib, upadacitinib, and tralokinumab, which could be used as an alternative option. As our knowledge of the endotypes of the disease expands, precision medicine will be applied for the best results. Omalizumab has been used in clinical trials and real‐life cases to treat CSU, but a debate on the efficacy of the monoclonal is frequently reported. However, EACCI′s current guidelines for the disease include omalizumab as an add‐on treatment for recalcitrant cases.

**Summary**
• The report includes current data on clinical trials related to the use of biologic agents in AD and CSU.

## 1. Introduction

Atopic dermatitis (AD) is a disease with increasing prevalence, a significant impact on patients’ lives, and a cost burden for the families [1]. Phenotypes and endotypes of the disease, as well as different responses to treatment, are currently investigated. A variety of monoclonal antibodies target cytokines implicated in the pathophysiology of the disease, including TH2, TH1, TH22, and TH17 subpopulations. These monoclonal antibodies have been used efficiently in other skin disorders, but direct application to AD was not possible [2].

Chronic spontaneous urticaria (CSU) is a common disease in the general population. Signs and symptoms of urticaria are related to the degranulation of mast cells and basophils with subsequent massive release of histamine, which produces the characteristic wheals and angioedema lasting for more than 6 weeks [3]. Autoimmunity is implicated in the etiology of the disease as well as cytokine dysregulation, including IL‐24. Omalizumab has been licensed as a treatment option for severe cases, and the monoclonal is FDA/EMA approved [3].

## 2. Methods

The study is a narrative review of available published data on the treatment of AD and CSU with monoclonal antibodies. The study period includes reports until the end of 2023. Data from different phase clinical trials, including RCTs, observational, meta‐analysis, and real‐world evidence, which were published in PubMed, are classified according to different parameters evaluating AD and CSU. The keywords included the scientific names of biological factors, AD and CSU. Review reports were excluded. However, a limitation of the study is that it reports data available in PubMed and no other search platforms. There was no statistical analysis due to the variety of parameters evaluated in each study. The strategy of the review was to record, analyze, and evaluate available published data.

Research studies use the same validation parameters for AD to have comparable results, which include the eczema score indexes, Eczema Area and Severity Index (EASI), Investigator’s Global Assessment (IGA), and SCORing body clearanceweeks. A significant percentage of (SCORAD) [2]. In addition, pruritus evaluation was accomplished using the peak pruritus score (NRS) and visual analog scale (VAS). Quality‐of‐life assessments include the Patient‐Oriented Eczema Measure (POEM), Dermatology Life Quality Index (DLQI), and Children’s Dermatology Life Quality Index (CDLQI). The parameters used for assessment of CSU were the Urticaria Activity Score (UAS), DLQI, VAS, Chronic Urticaria Quality‐of‐Life Questionnaire (CU‐Q2oL), and a dermatology‐specific questionnaire Skindex29, in addition to a separate assessment of hives, pruritus, erythema, and angioedema [3]. Mean value is defined as the average of the dataset.

## 3. Results

Dupilumab is an inhibitor of the common interleukin‐4(by IL‐4Rα/γc)/13 signaling pathway. Phase 2 trials detected improvement of inflammation in skin biopsies of the lesional and nonlesional atopic skin. Transcriptome analysis included known genes implicated in AD (Table 1). These initial studies encouraged clinical application. Clinical trials Phase 2 and Phase 3 measured the clinical effectiveness using different clinical parameters (Table 2). Dupilumab was administered in a loading dose of 400–600 mg and 300 mg/2 weeks or 4 weeks in adults suffering from moderate to severe AD. Follow‐up period in most reports was usually 16 weeks, but LIBERTY AD OLE extended the follow‐up period to 148 weeks. A significant percentage of patients vs. placebo improved the EASI75 score (59.13% vs. 19.03%), showing approximately a mean change of −78.55 vs. −40.62, meaning an improvement of 62.32% vs. 29.02%, in placebo. Regarding IGA ≤ 0‐1, 38.98% of patients vs. 12.08% in placebo improved with a mean value of −38.62 vs. −14.7 and SCORAD mean value of −52.75 vs. −23.55 in placebo. Pruritus was better in 42.03% of patients vs. 15.2% in placebo, with a mean change of −33.87, meaning an improvement of 52.42% vs. 21.35% in placebo. Quality‐of‐life parameters measured by NRS ≥ 4 (mean change 53.2 vs. 22.3), DQLI (mean change −8.27 vs. −4.56), and POEM (mean change −5.85) showed significant changes after treatment. Rescue treatment was more frequent in the placebo than in the dupilumab group (50% vs. 16%) [7]. The improvement sustained up to 52 weeks and even 3 years in different reports. Publications with combination treatment with steroids or calcineurin inhibitors reported better results [4].

**TABLE 1:** Reports measuring atopic dermatitis parameters and transcriptome in skin biopsies of patients in initial trials of dupilumab use in atopic dermatitis.

Publication	No. of patients	Duration	Dose	Measurements	Transcriptome
N Engl J Med. 2014 [4]	18	4 w M, +	1:1 M4B, 1:3 Pc: 150, 300	IgE, TARC, eosinophil counts	RNA expression Improvement 21%: 24%, 49%

J Allergy Clin Immunol 2019 [5]	54 Skin biopsies	16 w	1:1, 200Sc: Pc	Genes (IL‐13, IL‐31, CCL17, CCL18, and CCL26, epidermal hyperplasia, T‐cell and dendritic cell ICOS, CD11c, and CTLA4, T_H_17/T_H_22 activity and activity of epidermal genes)	Lesional/nonlesional skin 68.8%/110.08% vs. −10.5%/55%

**TABLE 2:** Clinical trials related to the use of dupilumab in atopic dermatitis.

Publication	No. of patients	Duration	Dose	Percentage of patients with reduction of EASI75/placebo	EASI/Pc	Percentage of patients with reduction of IGA to ≤ 0‐1/Pc	Clearance IGA ≤ 0‐1: Pc	Percentage of patients with pruritus/Pc	Pruritus/Pc	Quality of life
N Engl J Med. 2014 [4]	109	12 w M, +	1:1 M4A 1:4, 75: 150: 300: Pc M4B, 1:3, 150: 300: Pc	62%: 15%	EASI75 Mean −74: −23.3	Patients with IGA ≤ 0‐1 40%: 7%	IGA ≤ 0‐1 −49.5: −14.7		Pruritus reduction by 57.%: 15.1%	

	Combined	4 w	1:1 300/2 w: Pc	62%: 40%	EASI75 −75.6: −52.5	Patients with IGA ≤ 0‐1 52%: 30%	IGA ≤ 0‐1 Pc −52.5: −30.6		Pruritus/Pc −70.7: −24.7	

N Engl J Med 2016 SOLO 1 [6]	671	16 w	1:1:1 Loading 600, 300Sc/1 w: 300Sc/2 w: Pc	52%: 50%: 13%	EASI75 72.3%: 72%: 37.6%	Patients with IGA ≤ 0‐1 38%: 37%: 10%			NRS ≥ 4 50%: 50%: 25%	DLQI −9: −9.3: −5.3

SOLO 2	708		1:1:1 Loading 600, 300Sc/1 w: 300Sc/2 w: Pc	48%: 45%: 12%	EASI75 67.1%: 69.1%: 30.9%	Patients with IGA ≤ 0‐1 36%: 36%: 8%			NRS ≥ 4 50%: 45%: 15%	DLQI −9.5: −9.3: −3.6

Lancet 2016, LIBERTY AD CHRONOS [7] meta‐analysis	740	52 w, +	3:1:3; 300Sc/1 w: 300Sc/2 w: Pc	64%: 69%: 23%	EASI75 −77.3: −76.7: −43.2	Patients with IGA ≤ 0‐1 39%: 39%: 12%		NRS ≥ 4 51%; 59%: 20%	NRS ≥ 4 −25.6: −27.2: −19.8	DLQI −10.5: −9.7: −5.3

		54 w	3:1:3; 300Sc/1 w: 300Sc/2 w: Pc	64%: 65%: 22%			IGA ≥ 2 −40%: −36%: 13%	NRS ≥ 4 39%: 51%: 13%		DLQI −10.7: −10.9: −5.6

Br J Dermatol 2018 LIBERTY AD CAFÉ [8]	325	16 w, +	1:1:1; 300Sc/1 w: 300Sc/2 w: Pc	59.1%: 62.6%: 29.6%	EASI75 −78.2: −79.8: −46.6	Patients with IGA ≤ 0‐1 39.1%: 40.2%: 13.9%	SCORAD −58.3: −62.4: −29.5	NRS ≥ 4 77.8%: 87.6%: 44.2%	NRS ≥ 4 40.4%: 45.7%: 14.3%	DLQI −8.8: −9.5: −4.5

J Dermatol Sci 2018, Meta‐analysis [9]	2447	4–52 w	M, Sc300/1 w, Sc300/2 w: Pc		EASI75 Total −93 (1w): −86 (2w)		IGA ≤ 0‐1: Pc 3.82 (1 w): 3.23 (2 w)		Pruritus −0.81: −0.96	DLQI improvement

J Dermatol Sci 2019, LIBERTY AD SOLO 1 and LIBERTY AD SOLO 2 [10]	1379	16 w	1:1:1; 300Sc/1 w: 300Sc/2 w: Pc	50.2%: 47.7%: 13.3%	NLS −48.7: −47.4: −20.5 EASI75 −70.7: −70: −34.3	Patients with IGA ≤ 0‐1 36.8%: 37%: 9.3%		Pruritus 39.6%: 38.4%: 10.9%		POEM ≥ 4 63.4%: 69.4%: 25.4%

JAMA Dermatol 2020 LIBERTY AD SOLO‐CONTINUE [11]	422	36 w	2:1:1:1 Sc300/1 w and Sc300/2 w: Sc300/4 w: Sc300/8 w: Pc	EASI50 71.6%: 58.3%: 54.9%: 30.4%	EASI 50 −90.75: −85.35: −82.80: −72.73	Patients with IGA ≤ 0‐1 70.6%: 62.1%: 50%: 28.6%		NRS score ≥ 3 70%: 55.6%: 49.4%: 33.9%%	Pruritus −60.92: −51.61: −50.93: −27.05	DLQI 1: 4: 0.2: −3.1

JAMA Dermatol 2020 [12] LIBERTY AD ADOL	251	16 w, +	1:1:1 400 or 600 loading 200 or 300/2 w: 600 loading, 300/4 w: Pc	41.5%: 38.1%: 8.2%	EASI75 −65.9: −64.8: −23.6	Patients with IGA ≤ 0‐1 24.4%: 17.9%: 2.4%	SCORAD −51.6: −47.5: −17.6	Pruritus 36.6%: 26.5%: 4.8%	NRS −47.9: −45.5: −19	POEM −10.1: −9.5: −3.8

J Am Acad Dermatol 2020 [13] LIBERTY AD PEDS	367 children 6–11 y	16 w, +	1:1:1, 300Sc/4 w: 300Sc/2 w: Pc	69.7%: 67.2%: 26.8%	EASI50 ≥ 50%: ≥ 90% improve from baseline	Patients with IGA ≤ 0‐1 32.8%: 29.5%: 11.4%		Pruritus 50.8%: 58.3%: 12.3%	NRS ≥ 4 reduction	POEM improvement

J Am Acad Dermatol 2020, SOLO 1 and SOLO 2, AD ADOL, and CHRONOS [14]	1505	52 w	1:1 300/2w: Pc					Pruritus SOLO pooled 25.6%: 9.4% ADOL 24.4%: 9.6%	Pruritus SOLO pooled −47.5%: −20.5%; AD ADOL −47.9%: −19.0%; CHRONOS −57.3%: −30.9%	

Am J Clin Dermatol 2020 LIBERTY AD OLE [15]	2677	52 w (82.4%), 100 w (38.4%) 148 w (13%), +	300 mg/1 w Follow‐up 100 w: 148 w	91.3%: 96.6%	EASI75 −31.3: −29.2	Patients with IGA ≤ 0‐1 77.2%: 87.9%		Pruritus 79.1%: 75%	NRS −65.7: −65.4	Mean change POEM 5.4%

Br J Dermatol 2020 OLE extension [16]	38	Phase 2a, 12 w, 16 w, 52 w	A: Single dose 2 or 4 mg/kg at 1 w, 8 w: Baseline B: Baseline: 2 or 4 mg/kg/w at 8 w	A: 56% or 59%: 94%, B: 47%: 73% or 75%	EASI75 A: −76 or −73: −92, B: −63: −84 or −84	Patients with IGA ≤ 0‐1 A: 17% or 35%: 76%, B: 21%: 40% or 25%			Mean NRS A: 3 or 3: 2, B: 4: 3 or 3	

Allergy 2020, real world [17]	138	16 w	600 loading, 300/w	Improve 62%	EASI75 Mean change −73%			Pruritus Improvement 79%	NRS Mean change −53.5	DLQI Improvement 77.9% Mean change −9.2

J Eur Acad Dermatol Venereol 2021 LIBERTY AD PRE‐SCHOOL [18]	≥ 6 m to < 6 y, ≥ 6 m to < 2 years, 40 children	Phase 2,+/−, 4 w	Single dose 3 and 6 mg/kg at 4w: Pc	20% and 30%: 20% and 30%	EASI75 −43.2 and −22.4: −22.4 and −48.7		IGA ≤ 0‐1: Pc 44.5 and 30.85: 30.85 and 30:85		NLR 35.5% and 84.2%: 34.5% and 32.5%	

Br J Dermatol 2021 OLE extension [16]	≥ 6 to < 12 y 128 children	Phase 2 16 w, 12 and 16 w, 52 w follow up	A: Single dose 2 and 4 mg/kg at 8 w B: Single dose/w at 1 w: 8 w	A: 100% and 89% improve B 88%: 78%	EASI75 A: −85% and −84% Mean score at 1 and 8 w 35 and 29: 26 and 21	Patients with IGA ≤ 0‐1 A: 10% and 35% B: 35% and 47%: 44%	SCORAD A: −48 and −61, B: −43 and −67: −66	Pruritus A: 50% and 71%,	NRS A: −31% and −61, B: −38 and −66: −66	

Am J Clin Dermatol 2022 LIBERTY AD PED‐OLE [19]	≥ 12 to < 18, 294 children	52 w, +	2 or 4 mg/kg/w to 300/4 w	81.2% improvement	EASI75 −83.5 ± 23.5 improvement	Patients with IGA ≤ 0‐1 86.4% improvement	BSA −42.7 ± 26.2			CDLQI 86.4% ≥ 6 points improvement

*Note:* +, dupilumab + topical corticosteroids or cyclosporine.

Abbreviations: BSA, atopic dermatitis assessment; CDLQI, Children’s Dermatology Life Quality Index; DLQI, Dermatology Life Quality Index; EASI, Eczema Area and Severity Index; IGA, Investigator’s Global Assessment; NRS, peak pruritus score; POEM, Patient‐Oriented Eczema Measure; SCORAD, SCORing AD.

In children, dupilumab was used as a single dose of 2 or 4 mg/kg vs. placebo. Reports showed encouraging results with an EASI75 improvement in 51.6% or 75.55% of patients’ and a mean change of −70.1 or −65.85, respectively, to the dose used. Two studies extended treatment up to 52 weeks with a loading dose of 2–4 mg/kg and 6 mg/kg/4 w (OLE study, LIBERTY AD PED‐OLE study), and they report that 85% of patients improved, with an EASI75 score of −83.5 ± 23.5, while IGA ≤ 0‐1 improved in 84% of patients. Quality‐of‐life parameters evaluated efficacy up to 86.4% (CDLQI) and pruritus as an isolated characteristic by 50%–70% depending on the dose (Table 2). Reported studies confirm the effectiveness of dupilumab in both adults and children, examining all parameters (EASI75, IGA, pruritus, and quality of life) in 60% and 85% of patients, respectively.

Side effects (Table 3) included 420.4 adverse events/100 patient‐years. In our series, the total was 4.74%. Adverse effects were detected in 72.8% of patients, and 8.5 serious adverse events/100 patient‐years were detected. The most common were conjunctivitis 14.37% ranging from 2% to 35% in different reports, skin infection 23.9%, nasopharyngitis, headache, and infections, with the most frequent being herpes infection. Mild eosinophilia was also detected.

**TABLE 3:** Clinical trials with extended refer to the side effects of dulipumab.

	No of patients	Duration	Dose	Side effects	Conclusion
N Engl J Med. 2014 [4]	109	12 w M, +	1:1 Dupilumab: Pc	80%: 76% not serious, 2%:13% serious, 24%:5% skin infection, nasopharyngitis, headache	
Lancet 2017, LIBERTY AD CHRONOS [7]	740	52 w, +	3:1:3; 300Sc/1 w: 300Sc/2 w: Pc	83%:88%:84% not serious, 3%:4%:5% serious, 23.81%:16.36%: 9.42% conjunctivitis, 53%: 57%: 58%, local infections	
J Dermatol Sci 2018, Meta‐analysis [10]	2447	4–52 w	Sc300/1 w and Sc300/2 w: Pc	AE 42%: 43%	
Br J Dermatol 2018 LIBERTY AD CAFÉ [8]	318+	16 w	1:1:1; 300 Sc, 1 w: 300 Sc, 2 w: Pc	69·1%: 72·0%: 69·4% not serious, 1·8%: 1·9%: 1·9% serious, 15.5%:20.6%:16.7%, conjunctivitis	No safety concerns
J Dermatol Sci 2019, LIBERTY AD SOLO 1 and LIBERTY AD SOLO 2 [9]	1379	16 w	1:1:1; 300Sc/1 w: 300Sc/2 w: Pc	Exacerbation of AD, nasopharyngitis, conjunctivitis 31%:30%:30%, serious local reactions including herpes 5%:5%:4%	
British J of Dermatology 2020 SOLO 1 and 2 and CHRONOS [20]	1376 and 740	16/52 w M	1:1:1; 3:1:3, +; 1 w: 2 w: Pc	Decrease of neutrophils, platelets, and LDH. Increase of eosinophils < 1%	Absence of clinically important changes; no need for monitoring
JAMA Dermatol 2020 [9]	251	16 w	1:1:1:1; 200/2 w: 300/2 w: 300/4 w: Pc	9.8%:10.8%: 4.7% conjuctivitis, 8.5%:6%:18.8% local	
J Am Acad Dermatol 2020 [21]	1491	76 w	300/1 w	420.4 AE/100 patient‐years and 8.5 serious AE/100 patient‐years	Nasopharyngitis, conjunctivitis, and injection‐site reactions
Am J Clin Dermatol 2020, LIBERTY AD OLE [15]	2677	148 w	300/1 w	84.6% AE, nasopharyngitis, AD, upper respiratory tract infection, conjunctivitis (9.4%), headache, oral herpes, and injection‐site 9.6% severe, including flare of osteoarthritis, squamous carcinoma, flare of AD, syncope, hernia, appendicitis	
Allergy 2020 [17]	138	16 w	600 loading, 300/w	25%, eye irritation including dry eyes, itch, and tearing and 34% conjunctivitis, 10% headache, 5% injection‐site reaction, and 57% eosinophilia	
Am J Clin Dermatol 2021 [6]	12 to < 18 years	3 trials, LIBERTY AD ADOL: LIBERTY AD PED‐OLE: LIBERTY ASTHMA QUEST	Pc, 300/4 w, or 200/300/2 w: 2 or 4 mg/kg/w or 300/4 w: 200 or 300/2 w, Pc	(17/165; 10.3%): Pc (4/85; 4.7%) ≥ 1 event of conjunctivitis ADOL 12 (7.3%): 4 (4.7%), 12/275 adolescents (4.4%) serious at PED AD OLE (2/68, 2.9%) serious at QUEST	Atopic dermatitis trials had a higher incidence of conjunctivitis than in the asthma trials
LIBERTY AD ADOL		16 w	1:1:1 300/4 w: 300/2 w: Pc	10.3%:4.7% conjunctivitis, 7.3%: 4.7%, moderate or mild AE	
LIBERTY AD PED‐OLE			2 or 4 mg/kg/1 w: 300/4 w	3.4% serious AE, 4.4% conjuctivitis	
LIBERTY ASTHMA QUEST		52 w	200–300/2 w: Pc	2.9%: 2.6%, conjuctivitis	Lower incidents in asthma trials
J Eur Acad Dermatol Venereol 2021 LIBERTY AD PRE‐SCHOOL [18]	≥ 6 m to < 6 years, ≥ 6 m to < 2 years, 40	Phase 2,+/−, 4 w	Single dose 3 and 6 mg/kg	Anaphylaxis in the 3 mg group (+food allergy: peanuts, dairy, soya, egg, not related to the drug), mild or moderate 1 and 1 patient	Safe to ≥ 6 m to < 6 years patients comparable to older and adults
Am J Clin Dermatol 2022 LIBERTY AD PED‐OLE [19]	≥ 12 to < 18, 294	52 w, +	2 or 4 mg/kg/w	73.8% AE, 3.9/100 patients serious, 20.4% nasopharyngitis, 19% AD, 11.9% upper respiratory infection, 11.8% headache, 6.5% local, 8.8% conjunctivitis, 6.1% herpes virus	

*Abbreviations:* AD, atopic dermatitis; AE, adverse effects.

Omalizumab, an anti‐IgE antibody, has been used in different allergic diseases. There are not sufficient trials in the literature regarding the effectiveness of omalizumab in AD (12 single case studies, 15 case series, 5 prospective studies, and 2 small pilot randomized placebo‐controlled trials), and results are inconclusive. Based on retrospective studies, there is a considerable decrease of SCORAD to 20%–50% vs. 45%–80% in placebo or −71.2 vs. −31.5, respectively. On average, trials conclude that 62.42% are responders, while 16.88% are partial responders, and 20.7% are not responders (Table 4). Studies in children are encouraging, showing a benefit in 79% vs. 21% of patients and a considerable improvement of SCORAD, although bias could not be excluded. Overall, studies on the efficiency of omalizumab in AD show a range of responses to treatment.

**TABLE 4:** Clinical trials observing the use of Omalizumab in atopic dermatitis.

Publication	No of patients	Duration	Dose	Percentage of Patients with EASI improvement/Pc	Different parameters	Conclusion
J Am Acad Dermatol. 2005 [22]	3	4 m	450/4 w			No improvement

J Am Acad Dermatol 2006 [23]	7	7 m	Baseline: 375/2 w		IGA 3.75 ± 0.53: 0.5 ± 0.28	Preliminary case reports

J Allergy Clin Immunol 2007 [24]	11	5 m	150/2 w		SCORAD Improvement 18.8% of patients > 50%, 36.36% of patients, 25%–50%, 27.7% of patients < 25%	

Allergy Asthma Proc. 2008 [25]	21	9 m	150 or 300/2 w		Improvement	Concomitant asthma

Asian Pac J Allergy Immunol 2011 [26]	3	2–4 m	150 or 300/2 w	EASI75 Case 1, 50% from 12 to 6, Case 2, from 8 to 7, Case 3, 30% from 10 to 7	Itching score Case 1, decrease 43% from 7 to 3, Case 2, decrease 25% from 8 to 6, Case 3, decrease 50% from 9 to 5	

J Investig Allergol Clin Immunol 2011 [27]	11	10 m			SCORAD From 71.2 to 31.54 DLQI From 9.36 to 3.72	

J Am Acad Dermatol. 2013 [28]	7	12–67 m	225–300/2 w: Baseline		SCORAD 30.1: 75.4	

Clin Exp Dermatol. 2013 [29]	3	12 w	Case 1, 2 375/2 w Case 3, 300/2 w		SCORAD Case 1, from 78 to 40.7, Case 2, from 70.8 to 49.7, Case 3, from 73.9 to 14.1	

Clinical and experimental dermatology 2013 [30]	10	4 m	300/2 w: baseline		SCORAD 42.2 ± 9.0: 56.5 ± 8.4, improvement in 25.2% of patients VAS for pruritus 3.7 ± 1.3: 6.3 ± 1.7 DLQI scores 6.5 ± 3.7: 12.6 ± 3.7	

Allergy 2014, NCT01179529 [31]	20	28 w	150–300/2 w		SCORAD No change < 25% of patients, mostly FLG (+)	A particular subgroup of patients with AD can benefit from anti‐IgE

Clin Drug Investig 2015 [32]	9	10–78 m	150–450/4 w			Complete response 67%, partial 11%, nonresponders 22%

J Allergy Clin Immunol 2016, real life [33]	2RTCs, 13 case series, 103		600 mg/m			76% concomitant atopic asthma, 43% responders, 27.2% partial, 30.1% nonresponders

Int J Dermatol 2017, real life [34]	9		300/4 w			50% excellent response, 12.5% partial and 37.5% nonresponders

Review of the literature + retrospective studies	174 141	3 m–29.2 m	150–600/2 w: Pc		SCORAD Iyengar et al. Decrease to 20%–50%: 45%–80% Pozo et al. Decrease to 71.2: 31.5 DLQI Decrease from 9.36 to 3.72	74.1% complete response, 25.9% partial response, 78% responders, 14.18% nonresponders vs. 77% in treatment with cyclo

JAMA Pediatr 2020, ADAPT [35]	62 children 4–19 y	24 w, +	1:1 Dose depending on age and IgE levels: Pc	EASI75 32.8%: 38.4% of patients −12.7: −6.7	SCORAD −12.4: −5.1 DLQI 8: 12	Beneficial for children with moderate to severe AD

G Ital Dermatol Venereol 2019 [36]	214 patients, 12 single cases, 15 case series, 5 prospective, and 2 small pilot RCTs		150–600/2 w: Pc		79%; 21% of patients benefit	Probable bias

Rev Alerg Mex 2019 [37]	19 children	6 m	1:1 150–300/2 w: Pc		SCORAD Improved by 85.7%: 14%	Promising results in children

*Note:* +, omalizumab + topical corticosteroids or cyclosporine.

Abbreviations: DLQI, Dermatology Life Quality Index; EASI, Eczema Area and Severity Index; IGA, Investigator’s Global Assessment; SCORAD, SCORing AD; VAS = visual analog scale.

Janus kinase inhibitors, baricitinib (JAK1/2, Phase 2 completed), abrocitinib (PF‐04965842, JAK1, Phase 2), upadacitinib (JAK1, Phase 2), and ICB18424, LEO 124249/JTE‐052 (Phase 2) are under consideration [3]. Primary studies detected an EASI75 score improvement of 74%, 65%, and 64% and SCORAD‐24.34, −47, and −69.7 in patients treated with upadacitinib 30 mg/d, baricitinib 4 mg/d, and abrocitinib 200 mg/d, respectively, vs. EASI75 23%, 46%, and 15.4% and SCORAD −10.95, −21, and −29 in placebo. There was a considerable improvement up to 89% in pruritus scores with abrocitinib. The improvement in EASI was comparable to treatment with other monoclonals, showing efficacy of 71%, 65%, 61%, and 59% compared to placebo in patients treated with dupilumab 300 mg/4 w, abrocitinib 100 mg, nepolizumab 30 mg, and lebrikizumab 125 mg/4 w, respectively. However, nepolizumab, abrocitinib, and dupilumab show superiority in pruritus and quality‐of‐life parameters (11.69, 5.39, and 4.68 vs. placebo, respectively). Studies in children were not reported within the study period (Table 5).

**TABLE 5:** Clinical trials related to the use of upadacitinib and other Janus inhibitors in atopic dermatitis.

Publication	No. of patients	Duration	Dose	Percentage of patients with EASI75/Pc	EASI75	Percentage of patients with IGA ≤ 0‐1/Pc	Clearance, IGA ≤ 0‐1	Percentage of patients with pruritus	Pruritus	Quality of life
J Am Acad Dermatol 2019, Baricitinib NCT02576938 [38]	124	Phase 2, 16 w, +	4:3:3, 2 mg/d: 4 mg/d: Pc		EASI50 −64: −65: −46 Decrease by 65%: 46%		SCORAD −41: −47: −21			

J Allergy Clin Immunol 2020 Upadacitinib [39]	67	Phase 2, 16 w, monotherapy	1:1:1:1, 7.5:15:30: Pc		EASI75 39%: 62%: 74%: 23%		IGA ≤ 0‐1 35%: 59%: 75%: 19%			

Lancet. 2020, Abrocitinib [40]	263	Phase 2, 12 w	1:1:1:1:1 200:100: 30: 10 mg/d: Pc		EASI75 64.6%: 40.7%: 13.3%: 17.4%: 15.4% Mean change −47.4: −23.8		IGA ≤ 0‐1 43.8%: 29.6%: 8.9%: 10.9%: 5.8% Change −28.6: −20.2: −12.7: −7.4: −13.7 SCORAD −69.7: −49.2: −30.1: −26.7: −29		NRS score ≥ 4, 63.6%: 50%: 33.3%: 22.7%: 25.5% Mean change −25.4: −20.7:	

JEADV 2021 [41]	A; 3339 B; 1935	19 Phase 2 and 3 trials, 12–16 w	A; upadacitinib 30 mg: B; abrocitinib 200 mg: C; upadacitinib 15 mg: D; abrocitinib 100 mg: Pc		EASI75 A; 70%: 58%: B; 53%: 39%,		SCORAD50 A; 24.34: 10.95 B; −9.96: Pc D; −5.11: Pc		NRS4 B; improve 89%, D; improve 20% ≥ 98.2%: Pc	POEM A; −10.96: Pc B; −7.96: Pc DLQI (1752 patients) B; −5.46: −5.86 HADS A. (1852 patients) −2.75: −3.48, B; (1472 patients) −3.06: −2.30

	Vs other monoclonals 2193		A; dupilumab 300 mg+: Jak B: Dupilumab 300/4 w: abrocitinib 100 mg: Nemolizumab 30: Lebrikizumab 125/4 w: Pc	A; 45%, upadacitinib 30 mg and abrocitinib 200 mg > 97.5% probability of superiority to Dupilumab B; 71%: 65%: 61%: 59%: 56%		B; IGA ≤ 0‐1 (2217 patients) Dupilumab: Nemolizumab: abrocitinib 100 mg: Pc, 6.28: 4.38: 3.68: 3.68 SCORAD50 (1225 patients) abrocitinib 200 mg: Dupilumab 4.80: 4.73	NRS4 (1601 patients) Nemolizumab 30: abrocitinib 200 mg: Dupilumab 11.69: 5.39: 4.68, POEM (1798 patients) abrocitinib 200 mg: Dupilumab, −8.21: −7.04, DLQI scores (2026 patients) abrocitinib 200 mg: Dupilumab, −5.52: −4.62,			

*Note:* +: Janus inhibitor + topical corticosteroids or cyclosporine.

Abbreviations: CDLQI, Children’s Dermatology Life Quality Index; DLQI, Dermatology Life Quality Index; EASI, Eczema Area and Severity Index; HADS, Hospital Anxiety and Depression Scale; IGA, Investigator’s Global Assessment; NRS, peak pruritus score; POEM, Patient‐Oriented Eczema Measure; SCORAD, SCORing AD.

Lebrikizumab selectively targets IL‐13 and prevents formation of the IL‐13Rα1/IL‐4R. Lebrikizumab, in preliminary reports, showed an effect depending on the dose and interval between doses. Thus, the regimen of 125 mg/4 w or 250/2 w or 4 w was detected as the most effective compared to single doses. The EASI75 was improved in 57.75%, 63.73%, and 61.25% of patients in the different doses, and the improvement was 72.1%, 66.75%, and 70.55% accordingly, while improvement in IGA < 0‐1 was disappointing. Pruritus parameters showed an NRS improvement of 41.8%, 47.4%, and 70% in different doses vs. 27.3% in placebo; VAS of −60%, −42%, and −50% vs. 5% in placebo; and a considerable improvement in other quality‐of‐life parameters. Studies in children were not reported (Table 6).

**TABLE 6:** Clinical trials related to the use of lebrikizumab in atopic dermatitis.

Publication	No. of patients	Duration	Dose	Percentage of patients with EASI75/Pc	EASI75	Percentage of patients with IGA ≤ 0‐1/Pc	Clearance IGA ≤ 0‐1	Percentage of patients with pruritus/Pc	Pruritus	Quality of life
J Am Acad Dermatol 2018 (TREMBLE) [42]	209	Phase 2, 12 w+	1:1:1:1, 125, Single dose: 250, Single dose: 125/4 w: Pc	The single dose: not statistically important improvement 38.5%: 49.1%, 54.9%: 34%		Patients with IGA ≤ 0‐1 21.2%: 28.3%: 33.3%: 18.9%	SCORAD50 34.6%: 47.2%: 51%: 26.4%		VAS −34.9%: −32. 8%: −40.7%: −27.5%	DLQI −40%: −40%: −35%: −35%

JAMA Dermatol 2020 [43]	280	16 w, +	2:3:3:3 125 mg/4 w: 250 mg/4 w: 250/2 w: Pc 2 w	60.6%: 56.1%: 43.3%: 24.3%	EASI75 Improvement 72.1%: 69.2%: 62.3%: 41.1%	Patients with IGA ≤ 0‐1 44.6%: 33.7%: 26.6%: 15.3%:	IGA ≤ 0‐1 −18: −21: −38: −41	NRS 41.8; 47.4%: 70%: 27.3%	Pruritus −60%: −50%: −42%: +5%:	POEM −12.4: −11.4: −8.9: −5.8

JAMA Dermatol 2023 Adhere [43]	211	Phase 2I, 16 w, +	1:1 250/2 w: Pc/2 w	69.5%: 42.2%		Patients with IGA ≤ 0‐1 41.2%: 22.1%				

Br J Dermatol 2023 ADvocate1 and ADvocate2 [44]		52 w	1:1:1, 250/2 w: 250/4 w: Pc	78.4%: 81.7%: 66.4%	EASI75 Improvement 71.2%: 76.9%: 47.9%					

*Note:* +, lebrikizumab + topical corticosteroids or cyclosporine.

Abbreviations: EASI, Eczema Area and Severity Index; IGA, Investigator’s Global Assessment; SCORAD, SCORing AD; NRS, peak pruritus score; POEM, Patient‐Oriented Eczema Measure; DLQI, Dermatology Life Quality Index.

Tralokinumab in Phase 2 studies was not reported to be effective in different doses, while treatment presents a range of results. A Phase 3 study showed moderate effectiveness of the monoclonal in a dose of 300 mg every 2 or 4 weeks. EASI75 improvement was detected in 56·0% of patients vs. 35.7% in placebo and IGA ≤ 0‐1 in 38.9% of patients vs. 26.2% in placebo. In addition, SCORAD showed minimal improvement of −37.7 vs. −26.8 in the placebo. However, there was considerable improvement in quality‐of‐life parameters, including pruritus (mean score compared to the low dose −0.77: −0.14) and DLQI (85% vs. −8.8% in placebo). Studies in children were not reported. Preliminary studies are not encouraging for the use of tralokinumab in AD (Table 7).

**TABLE 7:** Clinical trials related to the use of tralokinumab in atopic dermatitis.

Publication	No. of patients	Duration	Dose	Percentage of patients with EASI75/Pc	EASI75	Percentage of patients with IGA ≤ 0‐1/Pc	Clearance IGA ≤ 0‐1	Percentage of patients with pruritus/Pc	Pruritus	Quality of life
J Allergy Clin Immunol 2018, NCT02347176 [45]	204	Phase 2b, 12 w	1:1:1:1 45: 150: 300/2 w: Pc	Dose 300: Pc 42.5%: 15.5%	EASI75 −13.67: −15.14: −15.72: −10.78	Patients with IGA ≤ 0‐1 11.6%: 19.5%: 26.7%: 11.8%	SCORAD Dose 300: Pc −9.42: −9.84		VAS Dose 45:300, −0.77: −0.14	DLQI Dose 300: Pc −3.51

J Allergy Clin Immunol 2019, EXTRA 1 and 2 [45]	204	Phase 2, 12 w, +	1:1:1:1, 45/2 w: 150/2 w: 300/2 w: Pc/2 w	25%: 11.7%: 33.2%: 11.2%		Patients with IGA ≤ 0‐1 15.8%: 7.1%: 22.2%: 10.9%	SCORAD −25.2: −14.7: −28.1: −14		NRS ≥ 4 20%: 10.3%: 25%: 9.5%	DLQI −7.1: −5: −8.8: −4.9

Br J Dermatol 2021, EXTRA 1 and 2 [46]	204	Phase 2b, 16 w	3:1, 300/2 w: Pc		EASI75 −15.72: −10.78		IGA ≤ 0‐1 −14.8			

Br J Dermatol 2021, EXTRA 3 [47]	380	Phase 2I, + 16 w and 32 w	2:1, 300/2 w: Pc and 300/2 w: 300/4 w	56.0%: 35.7% and 92.5%: 90.8%		Patients with IGA ≤ 0‐1 38.9%: 26.2% and 89.6%: 77.6%	SCORAD −37.7: −26.8		NRS Improvement 45.4%: 34.1%	DLQI Improvement 83.5%: 65.9% −11.7: −8.8

*Note:* +, tralokinumab + topical corticosteroids or cyclosporine.

Abbreviations: EASI, Eczema Area and Severity Index; IGA, Investigator’s Global Assessment; SCORAD, SCORing AD; VAS, visual analog scale; NRS, peak pruritus score; DLQI, Dermatology Life Quality Index.

Ustekinumab is a monoclonal antibody against IL‐12 and IL‐23. Ustekinumab 45 or 90 mg for 16 w showed remission in 50% of patients vs. 29% in placebo by the molecular analysis of the lesional skin. However, initial trials and real‐world data detected improvement of SCORAD measurements in 31% of patients vs. 19% in placebo and remission in 34.8% of patients with no effect in 34.8% of patients. Phase 2, RCT, case reports, and real‐world studies concluded that targeting the IL‐12/23 pathway is probably not a priority for patients with AD (Table 8).

**TABLE 8:** Clinical trials related to the use of ustekinumab in atopic dermatitis.

Publication	No. of patients	Duration	Dose	Molecular analysis	SCORAD50 U: Pc	Adverse effects	Conclusion
Exp Dermatol 2017 [48]	33	Phase 2, 16 w, +	1:1 45 mg, 90 mg: Pc At 0, 4, 16 w for patients weighing ≤ 100 kg or > 100 kg	50% U: 29% reversed K16 expression, reduction of K16 mRNA and a tendency of lower epidermal thickness, stronger decreases in mRNA at 4 wks T‐cell, Th2, and Th17 markers (MMP12, CX3CR1, CCL19, CCL17, PI3/elafin, lipocalin 2/LCN2) at 32 weeks decrease in (MMP12), T‐cells (GZMB), IL‐22/IL‐17‐induced S100 As, Th2‐attracting chemokines (CCL17, CCL18), Th1 (OASL) and Th17‐related products (CXCL1)	31%: 19%	24 self‐reported, no serious	
J Dermatology Treat 2018 [49], meta‐analysis	107	10 studies, 2 RCT, 8 cases			54.2% of patients improved	No	No significant difference in effect
J Dermatology Treat 2022, real world [50]	23				34.8% remission: 34.8% no effect		IL‐12/23 pathway is not an attractive target

Abbreviations: NM, not mentioned; Pc, placebo; RCT, randomized control trial; SCORAD, SCORing AD.

Nemolizumab, an IL‐31 inhibitor, is the 1st biologic to target IL‐31 with significant antipruritus effects. IGA ≤ 0‐1 and EASI75 improved in approximately 50% of patients with nepolizumab 30 mg sc every 4 weeks. However, pruritus and quality‐of‐life parameters showed significant improvement compared to placebo, for example, pruritus (mean change −67.3% vs. −35.8% in placebo) or POEM > 4 reached by 77.8% of patients, ameliorating symptoms by 40%–67%. However, studies are initial, and future clinical trials would provide more data regarding the efficacy of this monoclonal in AD (Table 9).

**TABLE 9:** Clinical trials related to the use of nemolizumab in atopic dermatitis.

Publication	No. of patients	Duration	Dose	Percentage of patients with EASI75/Pc	EASI75	Percentage of patients with IGA ≤ 0‐1/Pc	Clearance IGA ≤ 0‐1	Percentage of patients with pruritus/Pc	Pruritus	Quality of life
N Engl J Med 2017 [51]	264	Phase 2, 12 w	Sc 0.1: 0.5: 2 mg/kg/4 w: Pc	25%: 50%: 75%	EASI75 −23.0%: −42.3%: −40.9%: −26.6%		IGA ≤ 0‐1 −7.5%: −20.0%: −19.4%: −15.7%	VAS 25%: 50%: 75%	Changes −43.7%: −59.8%: −63.1%: −20.9%	Sleep −52.3: −59.1: −62.6: −31.9

J Allergy Clin Immunol 2018 [52]	215, 206	Part A 16 w Part B 52 w	2:1, Sc 60/4 w: Pc	25.9%: 12.5%	EASI75 Improvement 25.9%: 18.1%	Patients with IGA ≤ 0‐1 5.6%: 5.6%		VAS 34.3%: 13.9%	NLS ≥ 4 32.2%: 12.5%	POEM 73.2%: 44.4%

J Allergy Clin Immunol 2020 [53]	226	Phase 2, 24 w, +	Sc30/4 w: Pc	45.6%: 26.3%	EASI75 −68.8%: −52.1%	Patients with IGA ≤ 0‐1 36.8%: 21.1%	IGA ≤ 0‐1 change −40%: −20%		Pruritus −67.3%: −35.8%	DLQI mean change −10.5

N Engl J Med 2020 [54]	215	Phase 3, 16 w, +	2:1, Sc60/4 w: Pc		EASI75 −45.9%: −33.2%			VAS 67%: 50%	Pruritus mean change −42.8%: −21.4%	DLQI 40%–67%: 22%–50%

British Journal of Dermatology 2022 [55]	303	Phase 3, 52 w, +	2:1 Nemo/nemo Nepo/Pc Pc/Nemo	EASI75 By > 50%, 75% By > 75%, 59.7% By > 90%, 33.3%	EASI75 Decrease by 78.2%	IGA > 2 16.7% of patients		VAS: Baseline 23.1–31: 74.9–78.4 By > 50%, 58.3% By > 75%, 34.7% By > 90%, 13.9%	Decrease of pruritus by 65.9%	POEM > 4 77.8% of patients ISI > 4 Improvement 50.8%

*Note:* +, nepolizumab + topical corticosteroids or cyclosporine.

Abbreviations: DLQI, Dermatology Life Quality Index; EASI, Eczema Area and Severity Index; IGA, Investigator’s Global Assessment; ISI, Insomnia Severity Index; NRS, peak pruritus score; POEM, Patient‐Oriented Eczema Measure; VAS, visual analog scale.

A Phase 2 trial with fezakinumab monotherapy every 2 weeks for 10 weeks detected a decline in SCORAD 13.8 ± 2.7 in patients compared to 8.0 ± 3.1 in the placebo group that was stronger in the more severe forms (21.6 ± 3.8 vs. 9.6 ± 4.2 at 12 weeks and 27.4 ± 3.9 vs. 11.5 ± 5.1 at 20 weeks). The body surface area clearance at 12 weeks was 12.4% ± 2.4 vs. 6.2% ± 2.7 in the placebo group. Results are initial, but they emphasize that targeting IL‐22 would not influence AD signs and symptoms (Table 10).

**TABLE 10:** Initial trials report measuring atopic dermatitis parameters and transcriptome in skin biopsies of patients treated with fezakinumab.

Publication	No. of patients	Duration	Dose	Molecular analysis SCORAD50 U: Pc Adverse effects (AE)	Conclusion	
J Allergy Clin Immunol 2019, NCT01941537 [56]	59	Phase 2, Skin biopsies, 4 and 12 w	2:1 Loading 600, 300: Pc	Transcriptome improvement 82.8% and 139.4% vs. 39.6% and 56.3% ↓TH1/CXCL9, TH2/CCL18/CCL22, TH17/CCL20/DEFB4A, and TH22/IL‐22/S100A genes ↓ Inflammation indexes, (MMP12), T‐cell activation (ICOS and CD86/CD28), innate immune responses (MX1 and CXCL8), and molecules associated with TH1 (IRF1, CXCL9, CXCL10, and CXCL11), TH2 (CCL13, CCL17, CCL18, and CCL22), TH17 (CCL20, PI3/elafin, CXCL1, and IL36G), and TH17/TH22 activation (S100A7, S100A8, and S100A12) Higher expression of fingerprinting in IL‐high, 229.9% and 234.5% at Weeks 4 and 12 compared to low 254.6% and 2117.7% 1^st^ report showing a profound effect of IL‐22 blockade on multiple inflammatory pathways in AD		

J Am Acad Dermatol 2018 [57]	60	Phase 2, 12 w and 20 w, monotherapy	2:1, loading 600, 300/2 w: Pc		SCORAD 13.8 ± 2.7: 8.0 ± 3.1 at Week 12 18.8 ± 2.9: 11.7 ± 3.9 at Week 20 Improvement −24.4%: −14.7% and −34.1%: −23%	IGA −0.6: −0.3 and −0.9: −0.6 Improvement 15%: 5% and 25%: 15%

Recalcitrant cases of CSU are treated with omalizumab. It is more effective in the dose of 300 mg/2 or 4 w with complete responders 70.50% reaching up to 99.1% in some reports. However, a percentage of approximately 7% are not responders and a percentage reaching 32.63% relapse after discontinuation of the medication. UAS score was 11.3 vs. 25.36 in placebo (responders 61.9% vs. 16.6%), itching −8.04 vs. 7.8 (responders 65.63% vs. 35.1%), wheals −7.9 vs. −7.3 (responders 78% vs 27.7%), angioedema −24.4 vs. −22.8, DLQI 6.016 vs. 6.52 (responders 34.25% vs. 45.43%), implying a significant improvement in the skin assessment scores, but not in the quality‐of‐life scores. Few reports in children and adolescents evaluate the monoclonal as efficient in the treatment of the disease (Table 11).

**TABLE 11:** Clinical trials related to the use of biologic factors in chronic spontaneous urticaria.

Publication	No of patients	Dose	Duration	Improvement in different scales
J Allergy Clin Immunol 2008 [58]	12, autoimmune CU	150/4 w: baseline	16 w	UAS 7.50 ± 1.78: 2.66 ± 3.31, 66.5% of patients 50% improvement Rescue medication 99.3 tab: 29.8 tab in placebo DLQI 15: 7.5

J Allergy Clin Immunol 2011 [59]	49	A. Baseline: 75–375 mg/2 w B. 75–375 mg/4 w: Pc	24 w	UAS7 Mean change = 17.8: 7.9 A. 24.6 ± 7.4: 6.8 ± 10.0 B. 21.3 ± 7.6: 15.5 ± 11.0 Hives −9.2: −3.3 Decrease of symptoms 70.4%: 4.5% Pruritus Decrease of symptoms 59.3%: 9.1% Erythema Decrease of symptoms 66.7%: 18.2% Angioedema Decrease of symptoms 77.8%: 36.4% IGA 59%: 14% DLQI Improvement 62.4%: 15.3% Mean change = 4.8 Skindex29 Improvement 50.0%: 6.3% Cu‐Q2oL Improvement 53.2%: 5.9% Complete resolution 67%: 4%

J Allergy Clin Immunol 2011, MYSTIQUE [60]	90	Phase 2 1:1:1:1 75:300:600:Pc		UAS7 −9.8: −19.9: −14.6: −6.9 Improvement 4.4%: 36%: 28.6%: Pc Itch score −4.5: −9.2: −6.5: −3.5 Hive score −5.3: −10.7: −8.1: −3.5

Journal of Dermatology 2012 [61]	12	150–375 mg/2 w or 4 w: baseline	10–20 m	UAS 34.4 ± 6.8: 2.9 ± 1.5 Retreated UAS –29.5 ± 9.6: 34.3–24.7 CU‐Q2oL 57.5 ± 13.8: 10.3 ± 10.4 Pruritus 9.0 ± 0.8: 2.7 ± 0.8 Swelling 6.8 ± 3.1: 2 ± 0.01 Responders 100% Relapses 43.4%: 45.1%

N Engl J Med 2013 [62]	Phase 3, 323	1:1:1:1 75: 150: 300/4 w: Pc	12 w	UAS7 ≤ 6 27%: 43%: 66%: 19% Itch score −5.9: −8.1: −9.8: −5.1 Angioedema free 93.5: 91.6: 95.5: 89.2 days DLQI −7.5: −8.3: −10.2: −6.1

J Allergy Clin Immunol 2013 [63]	Phase 3, 336	3:1 300/4 w: Pc	(12 w), 24 w Mean duration 22.4 w: 20.6 w	UAS 7 −19.0: −8.5 in 52% of patients: 12% Itch score −8.6: −4.0 in 69.8% of patients: 39.8% Hives −10.5: −4.5 DLQI: baseline −9.7: −5.1 Angioedema free 91.0% of patients: 88.1% CU‐Q2oL −29.3: −16.3

Ann Allergy Asthma Immunol 2014 [64]	68	150/4 w: baseline	18 m	UAS7 32.2: 5.7 34 patients UAS7 improvement 26.5 points Mean medication baseline: 18 m 12.8: 2.2 Complete remission and time of remission 1 m (17.9%), 3 m (51.9%), 6 m (60%), 18 m (71.4%) Responders 79% Partial responders 18% Nonresponders 8%

J Allergy Clin Immunol 2016 ASTERIA I [65]:	318	1:1:1:1 Phase 3 75, 150, or 300 mg/4 w: Pc	24 w	UAS7 ≤ 6 30%: 36%: 62%: 25% Complete responders 23%: 20%: 48%: 13%

ASTERIA II	322	1:1:1:1 Phase 3 75, 150, or 300 mg/4 w: Pc	12 w	UAS7 ≤ 6 27%: 43%: 66%: 19% Complete responders 16%: 22%: 44%: 5% Median time of response 8 w: 7 w: 3 w

GLACIAL	335	3:1 Phase 3 300/4 w	6 m	UAS7 ≤ 6 56%: 12% Complete responders 43%: 4% Median time of response 6 m

J Eur Acad Dermatol Venereol 2017 [66]	158		24 m	UAS7 Mean change 15.6 VAS mean change 6.1 CU‐Q2oL mean change 29.6 DLQI score mean change 7.7 UCT mean change 8.3 Absenteeism/w mean = 3.7%, 20.6% loss of productivity

Curr Med Res Opin. 2018 [67]	88	150–300: baseline	24 m	Hives 71.6%: 83% Itching 38.6%: 56.8% Swelling 18.2%: 45.5% Responders 77.8% Complete response 47% Nonresponders < 10%

J Allergy Clin Immunol 2018 [68]	43 trials			Effective for symptomatic dermographism, cold urticaria, and solar urticaria

J Allergy Clin Immunol 2018, XTEND‐CIU [69]	205	300/4 w: Pc	48 w	DLQI 19.8%: 66% Angioedema 0.8%: 7.3% Relapses 21%: 60.4%,

J Am Acad Dermatol 2018 [70]	31	300 mg/4 w	24 w	Improvement ≥ 90%

Expert Opin Biol Ther 2018 [71]	1507 P, 84 studies CIU = 25.0%, with comorbidities = 60.7%, Autoimmune = 3.6%	150 or 300 mg/2 or 4 w		Overall disease activity decreased in 82.4% of patients UAS 5.6 ± 0.8, mean change in 82.4% of patients UAS7 29.2 ± 7.2, mean change in 87.5% of patients UCT 6.2 ± 2.0, mean change in 113.5% of patients DLQI 21.6 ± 5.0, mean change in 81.6% of patients CU‐Q2oL 66.1 ± 17.9, mean change in 68.1% of patients Overall quality of life Improvement in 76.2% of patients Responders 85% Nonresponders 15% Complete response 67.9% Partial response 21.6% Refractory 10.5%,

Allergol Int 2018 [72]	25		10 m	Responders 58%, At 6^th^ month 61% Relapse 91% and retreated Anti‐TPO, AutoAbs(+) in partial responders

Ann Allergy Asthma Immunol. 2018, AIFA [73]	322	300/4 w: Baseline	24 m	UAS7 –24.6 ± 9.9 Itch score −12.3 ± 5.8 Responders 66.7% Relapses 40.8%

	15–65: ≥ 65 y (9.9%)			UAS7 −24.4: −26.3 In 84.2%:83.3% of patients Itch score −12.2: −13.3 In 67.8%: 56.7% of patients

Drug Des Devel Ther 2019 [74]	32	300/4 w: baseline	6–13 m (46 w)	UAS 4: 29.5 DLQI 20: 5

N Engl J Med 2019, NCT02477332 [75]	382	Ligelizumab 24: 72: 240 Omalizumab 300: Pc	Phase 2, 12 w	Improvement of symptoms 30%: 51%: 42%: 26%: 0% Control of symptoms 30%:44%:40%: 26%: 0% UAS 28.6%: 31.7%:30.3%: 29.3%:31.1%

Allergol immunopathology (Madr) 2020 [76]	29 adolescents	300/4 w: Baseline	12 w	UAS 13:2 (12 w) Responders, 58% complete response 12 w, Mean time of response 7.5 w Responders up to remission 89.6% complete Partial responders 6.9% Nonresponders 3.5% Relapse 13%

Dermatologic therapy. 2020 [77]	6 children, adolescents	150–300/m	5–12 m	Responders 91.66% Partial responders 8.33% Relapse 16.6%

Pediatric dermatology. 2020 [78]	19 children and adolescents	300 mg/4 w	2–24 m	Responders 84% Partial responders 3% Nonresponders 3%

Allergy 2020, EACCI [79]	1620	300/4 w		UAS7 Mean change −11.05 (12.87 to −9.24) DLQI Mean change −4.03 (−5.56 to −2.5)

Pediatr allergy immunol. 2023, NCT03437278 [80]	49	Phase 2, 2:1:1 ligelizumab 24 mg/4 w:120 mg/4 w: Pc	24 w	UAS7 20.4 ± 13.0: −22.5 ± 13.5: −21.3 ± 14.5 Mean change from baseline −15.7 ± 10.9: −18.4 ± 12.3: −13.0 ± 13.0 Improvement 61.5%: 33.3%: 33.3%

Abbreviations: CU‐Q2oL, Chronic Urticaria Quality of Life Questionnaire; DLQI, Dermatology Life Quality Index; Skindex29, a dermatology‐specific questionnaire; UAS, Urticaria Activity Score; UCT, University of Cape Town; VAS, visual analog scale.

## 4. Discussion

AD is a chronic disease characterized by inflammation of the skin accompanied by pruritus, which seriously affects well‐being. The prevalence is reported to affect 1%–20% of the adults [4] and 0.2%–24.6% of the adolescents [81]. Chronic disease is moderate to severe in 20% to 1/3 of patients [12]. The etiology of the disease is a genetically determined disruption of the skin barrier accompanied by TH2 inflammation of the epidermis. Affected skin predisposes to infections [3]. Patients have a deterioration of quality of life with high estimates of depression and anxiety. The atopic march is related to other allergies, mainly allergic rhinitis and asthma (60% of patients) [14]. In severe cases, aggressive treatment with UV phototherapy and immunosuppressants is mandatory, including cyclosporin (53%–95% effective), methotrexate (42%), and azathioprine (39%) [8]. The use of monoclonal antibodies to target the inflammation shows that the disease involves multiple cytokine pathways (see Table 12).

**TABLE 12:** The monoclonal antibodies used in atopic dermatitis, their targets and type.

Biologic factor	Target	Antibody	Strength of evidence	Route of administration	FDA/EMA approved
Dupilumab	TH2 inflammation inhibits interleukin‐4 (by IL‐4Rα/γc) and interleukin‐13 signaling (by Type II, IL‐4Rα/IL‐13Rα)	Recombinant human IgG4 monoclonal antibody	A	Sc	FDA and EU approved. Approved also for asthma, chronic sinusitis, EoE

Omalizumab	Blocks IgE immunoglobulin and binds to its receptor FcεRI	Humanized monoclonal antibody	C	Sc	Approved for asthma. Used in trials in AD and food allergies

Ustekinumab	Blocks IL‐12 and IL‐23 by targeting the common p40 subunit inhibiting Th1 and Th17/Th22 responses	Fully human IgG1κ monoclonal antibody	B	Sc	Approved for inflammatory bowel diseases, psoriasis, and psoriatic arthritis

Nemolizumab	Binds to interleukin‐31 receptor A (IL‐31RA)	Humanized monoclonal antibody	B	Sc	Not approved Approved in Japan

Upadacitinib	Selective and reversible Janus kinase (JAK) inhibitor (JAK1 or JAK1/3)		B	Prolonged released tablets	EU authorized for AD, psoriasis, rheumatoid arthritis, and inflammatory bowel disease

Lebrikizumab	Selectively targets IL‐13 and prevents formation of the IL‐13Rα1/IL‐4R	Humanized monoclonal antibody	D	Sc	Not approved

Tralokinumab	Specifically binds to the Type 2, IL‐13 prevents from binding to receptor	Fully human IgG4 monoclonal antibody	D	Sc	EU authorized for AD

Fezakinumab	IL‐22 blocking	Recombinant monoclonal antibody	D	IV	Not approved

*Note:* Also strength of evidence, route of administration, and FDA/EMA approved.

Similarly to our results, Dupilumab, a monoclonal antibody that targets the IL‐4/13 receptor, showed an effectiveness of up to 40%–60% of AD signs in adults [17], as well as SCORAD, pruritus, and quality‐of‐life parameters. Real‐world studies emphasize that although signs of the disease may persist, significant amelioration of quality‐of‐life parameters, including pruritus, are important in everyday practice [17]. Results are better with combined treatment with local steroids or calcineurin inhibitors especially in the younger population (≤ 12 years). Studies in children are encouraging, and they detected an improvement with a single dose of 2 or 4 mg/kg, as EASI75 was detected in 75.5% of patients with a mean change of −65.85. The regular administration of the drug in a dose of 6 mg/kg/4 w was quite effective. An EASI75 score of‐ 83.5 was reported in 85% of patients approximately, and IGA ≤ 0‐1 in 84% of patients, a considerable improvement of pruritus in 50%–70% of patients and quality‐of‐life parameters. Responders show a rapid, robust, and significant improvement of AD signs and pruritus [82] lasting for a prolonged time (> 52 weeks). The above findings support the hypothesis that TH2 inflammation has a significant role in the etiopathogenesis of AD. The safety profile of dupilumab has the disadvantage of conjunctivitis in 14.37% of patients, skin infection in 23.9% of patients, nasopharyngitis, headache, and eosinophilia, with serious events reported as 8.5 serious adverse events/100 patient‐years.

According to the literature and our results, omalizumab would not be the better choice in patients with AD [22]. However, many studies treating patients, for example, for asthma, detected a serious improvement in concomitant AD. Case reports and real‐life studies showed a considerable number of long‐term responders up to 62%, while results are more encouraging in children (80% responders).

JAK inhibitors started to be popular after the first reports. They are administered locally or orally, and they target a number of cytokines by inhibiting the JAK 1,2/STAT pathway of inflammation. Thus, they block upstream the intracellular signaling of many cytokines implicated in AD. Different AD endotypes show a predominance of TH2, TH22, TH1, and TH17 inflammatory pathways, and blocking multiple cytokine axes is considered more effective [38]. Upadacitinib shows the highest efficacy in the dose of 30 mg/daily with 83.6% of patients reaching EASI50, compared to upadacitinib 15 mg with an EASI50 in 70.5% of patients. The same study compared EASI score in patients treated with abrocitinib 200 mg and with dupilumab 300 mg/2 w, and it detected an EASI50 improvement in 74.5% and 63.4% of patients, respectively [41]. In addition, rare adverse events (headache, elevated creatinine phosphatase, and nasopharyngitis) and convenient administration are the main advantages of JAK inhibitor treatment.

Anti‐IL‐13 treatments have a central role in TH2 inflammation, and gene polymorphisms are associated with predisposition to AD. The increased mRNA for IL‐13 in lesional skin is correlated with the severity of the disease [42]. Starting with lebrikizumab, which selectively blocks soluble IL‐13, thus preventing binding to the IL‐13Ra/IL‐4Ra receptor, heterodimerization, signaling, and downregulation of important skin barrier components, is a promising alternative [42]. In our study, there was a significant improvement of all parameters examined, giving an EASI75 of approximately 70% in 65.8% of patients, clearance remission up to 39%, and a significant improvement of pruritus score (mean change −50.35 vs. 16.25). Regarding tralokinumab, a dose‐dependent effectiveness shows that 150 or 300 mg/2 w is probably the appropriate dose [43]. Although there is a moderate improvement in AD signs, the monoclonal offers significant improvement in quality‐of‐life measurements. However, studies are in Phase 2, and more data are required to prove the efficacy of this monoclonal antibody. Lebrikizumab and tralokinumab would preferably target the TH1 neutrophilic endotype.

Blocking the IL‐31, the pruritus cytokine, inhibits the development of chronic disease by regulating the transcription program related to the elongation of sensory nerve stimulation and response to minimal stimuli that promotes the “itch‐scratch cycle.” Apart from pruritus, IL‐31 has anti‐inflammatory effects. Nepolizumab in a dose of 30 mg or 60 mg sc/4 w in Phase 2 trials was considered effective. The administration showed moderate improvement of clearance of dermatitis measured by EASI75 and IGA, although results vary between trials. However, pruritus scales showed improvement up to 65% as well as quality‐of‐life scores. Side effects show a paradox of worsening of AD signs rarely in patients, although they have an improvement of VAS [54].

Preliminary results blocking IL‐22 with fezakinumab were disappointing. The drug is related to the TH2 subpopulation, which acts on IL‐20, IL‐22, and IL‐10, a homeostatic protein. IL‐10 and IL‐17 are the main drivers of keratinocyte proliferation with hyperplasia and the initiation of barrier defects. Targeting IL‐22 circumvents many other inflammatory cytokines that are related to the etiology of inflammation in AD, and preliminary results showed that it would not be effective in AD disease [56].

CSU has a lifetime prevalence of approximately 0.5%–1% [63] and affects 3% of children [71]. Regarding the etiology, 45% of adults express an IgG antibody directed against the a‐subunit of a mast cell receptor, leading to mast cell and basophil activation. Histamine release leads to c5 complement chemotactic activity with recruitment of neutrophils and eosinophils seen in skin biopsies. It is reported that 50% of adults and 90% of children respond to antihistamines [60, 61, 71]. Moreover, 66.7% of patients would have a rescue treatment with steroids in addition to conventional treatment with antihistamines [78]. A number of options would control these cases, such as hydroxyzine, H2‐receptor antagonists, and cyclosporine. However, remission is not reproducible. Omalizumab induces a rapid reduction in free IgE levels and a decrease in FCeR1 expression on mast cells [57]. It is FDA and EU approved for recalcitrant cases of CSU.

Our data show that omalizumab would have some benefit in a number of patients, although relapses are also frequent. Doses of 150 mg or 300 mg every 4 weeks within a course of 12–24 weeks would have similar results [71]. Remission would achieve 49%–90% of patients in different studies. UAS score improved rapidly within the 1st week in 2/3 of patients [59]. Late responders would have a remission within 8–10 weeks. It was frequent to have relapses in responders after a course of 24 weeks, within 5–16 weeks. In these cases, a 2nd or 3rd course was an acceptable practice [65], raising questions about the appropriate duration of treatment [60]. After 6 months, 61% of patients relapse, but a percentage of 91% respond to a 2nd 24 weeks course. Compliance was usual due to no serious adverse effects. EACCI becomes skeptical regarding overall efficacy, although current guidelines would apply the monoclonal in recalcitrant cases of CSU [79]. In the literature, omalizumab was described to be effective in dermographism (86% complete response), cold urticaria, delayed urticaria (60% complete response), cholinergic urticaria (62% complete response), and solar urticaria (80% response) [67]. Other monoclonal antibodies, such as ligelizumab, are under investigation [74, 79]. Initial trials with the monoclonal ligelizumab compared its effectiveness to omalizumab, and they detected a response of symptoms in 50% of patients without significant side effects.

## 5. Conclusion

The use of monoclonal antibodies to treat AD seems tricky due to the fact that AD activates different pathways of inflammation, and a number of cytokines are implicated. Monoclonal antibodies in Phase 2 and Phase 3 clinical trials could ameliorate different parameters of AD. Targeting the IL‐4/13 pathway with dupilumab shows a complete clearance in 40%–60% of cases and significant changes in children. However, some patients experience serious side effects, such as conjunctivitis. JAK/STAT inhibitors are under consideration because of convenient administration. They are quite promising monoclonals due to the fact that they block many pathways of inflammation upstream. Preliminary results in the use of anti‐13 biologics and probably anti‐IL‐31 are also a promising option. Results regarding the use of monoclonals in AD are preliminary, but quite promising, and they could ameliorate the symptoms of patients suffering from moderate and severe disease. Omalizumab, although not useful in AD, is effective in recalcitrant cases of CSU with frequent relapses, which make skeptical assessments for the treatment.

## Funding

No funding was received for this study.

## Conflicts of Interest

The author declares no conflicts of interest.

## Data Availability

The data that support the findings of this study are available from the corresponding author upon reasonable request.
